# Effect of Intraoperative Hypotension on Survival After Resection of Colorectal Liver Metastases

**DOI:** 10.1155/1992/19395

**Published:** 1992

**Authors:** B. Nordlinger, P. Balladur, E. Delva

**Affiliations:** 1 Department of Surgery Hôpital Saint-Antoine 184 Faubourg Saint-Antoine Paris 75012 France; 2 Department of Anesthesiology Hôpital Saint-Antoine 184 Faubourg Saint-Antoine Paris 75012 France


perform than cystgastrostomy but had a slightly lower rate of postoperative complications including bleeding[4]; the cyst recurrence rate was virtually identical (8 per cent versus 10 per cent). The same tendency towards a higher complication rate after cystgastrostomy emerges from three previous reports[6–8].

In summary, cystgastrostomy is an appropriate procedure for 'mature' acute pseudocysts that are large enough to cause symptoms but not so large as to prevent adequate drainage by this approach. For the rest (probably the majority), cystjejunostomy offers a safer alternative at the expense of a longer operation.

### REFERENCES

1. Yeo, C.J., Bastidas, J.A., Fishman, E.K., Zinner, M.J. and Cameron, J.L. (1990) The natural history of pancreatic pseudocysts documented by computed tomography. *Surg. Gynecol. Obstet.*, **170**, 411–417
2. D'Egidio, A. and Schein, M. (1991) Pancreatic pseudocysts: a proposed classification and its management implications. *Br. J. Surg.*, **78**, 981–984
3. Vansonnenberg, E., Wittich, G.R. and Casola, G. *et al.* (1989) Percutaneous drainage of infected and noninfected pancreatic pseudocysts. *Radiology*, **170**, 757–762
4. Steiner, E., Mueller, P.R. and Hahn, P.F. *et al.* (1988) Complicated pancreatic abscesses: problems in interventional management. *Radiology*, **167**, 443–446
5. Newell, K.A., Liu, T., Aranha, G.V. and Prinz, R.A. (1990) Are cystgastrostomy and cystjejunostomy equivalent operations for pancreatic pseudocysts? *Surgery*, **108**, 635–639
6. Frey, C.F. (1978) Pancreatic pseudocysts — operative strategy. *Ann. Surg.*, **188**, 652–662
7. Schattenkerk, M.E., Vries, J.E., Bruining, H.A., Eggink, W.F. and Obertop, H. (1982) Surgical treatment of pancreatic pseudocysts. *Br. J. Surg.*, **69**, 593–594
8. Kohler, H., Schafmayer, A., Ludtke, F.E., Lepsien, G. and Peiper, H.J. (1987) Surgical treatment of pancreatic pseudocysts. *Br. J. Surg.*, **74**, 813–815

S. Paterson Brown
and R. C. N. Williamson
Royal Postgraduate Medical School
Department of Surgery
Hammersmith Hospital
Du Cane Road
London W12 0HS, UK

### EFFECT OF INTRAOPERATIVE HYPOTENSION ON SURVIVAL AFTER RESECTION OF COLORECTAL LIVER METASTASES

### ABSTRACT

*Younes, R.N., Rogatko, A. and Brennan, M.F. (1991) The influence of intraoperative hypotension and perioperative blood transfusion on disease-free survival in patients with complete resection of colorectal liver metastases. Annals of Surgery,* **214**, *107-113.*

**An increased interest in surgical treatment of liver metastases from colorectal origin has evolved recently. However not all patients benefit from this approach, with early recurrence and death still being encountered. To evaluate clinical as well as perioperative factors that might significantly affect the outcome of patients with completely resected colorectal liver metastases, we examined 116 patients who underwent resection between September 1987 and August 1989. Median follow-up time was 13.2 months (0.6 to 31.4 months). The overall survival rate was 91% at 1 year and 75% at 2 years. Median survival was not reached. Median disease-free survival time was 11.5 months, with 49.4% and 21.2% of the patients being free of disease at 1 and 2 years, respectively. By univariate analysis, site of primary colorectal cancer, preoperative carcinoembryonic antigen (CEA) level, size of metastases, number of metastases, length of operation time, percentage mean arterial pressure, number of hypotensive episodes, duration of hypotensive episodes, and whole blood transfusion significantly affected recurrence rate following resection. However only site of primary tumor, CEA, number of metastases, and number of hypotensive episodes remained significant in the multivariate analysis. The most significant single factor that affected recurrence rate was the number of hypotensive episodes during the operative procedure. It is concluded that hypotensive episodes, even when well controlled, should be avoided during operation to maximize the chances of cure and prolong disease-free survival of patients with colorectal liver metastases.**

### PAPER DISCUSSION

KEY WORDS: Liver resection, liver metastases, intraoperative hypotension

The aim of this paper was to evaluate pre- and perioperative factors that could affect disease recurrence and survival in patients undergoing complete hepatic resection for colorectal liver metastases. The factors which significantly affected the recurrence rate following resections in multivariate analysis were the site of the primary tumor, the CEA level before resection, the number of metastases and the number of hypotensive episodes during surgery.

The authors conclude that hypotensive episodes should be avoided during the resections of liver metastases in order to improve the chances for cure. This message is original.

This study reports an homogeneous series of more than one hundred patients originating from one institution and operated upon during a short period of time (2 years). However the duration of follow-up was short, ranging from 0.6 to 31.4 months (median 13.2). This can jeopardize the conclusions since it is well known that many recurrences after resection of colorectal liver metastases occur after the first year of follow-up and are not taken into account in this series. In the multicenter study of Hughes[1] recurrences were observed in 70% of 607 patients who had undergone curative resections, but only half of these recurrences (36% of total patients) were observed during the first year of follow-up. Thus the conclusions of the present paper should relate only to early recurrences.

Preoperative CEA level is a well recognized prognostic factor. However it is more surprising that the grade of the primary cancer did not affect survival and on the other hand that the site of the primary cancer was a statistically significant factor in multivariate analysis. This is contrary to other recent large studies[2-3] and may be related to the relatively small number of patients included in the study and to the short follow-up.

The major interest of this paper is related to its results concerning the role of perioperative factors. The most significant factor that affected recurrence rate was the number of hypotensive episodes during the operative procedure and one of the conclusions was that hypotensive episodes should be avoided to minimize the risk of recurrences. This conclusion raises several comments. Baseline MAP was defined as the pressure when the surgeon started the operation but this period is frequently associated with important variations in the arterial pressure. Conversely hypotension was defined as a decrease of 20% of baseline MAP. For a baseline value of 140 mm Hg, 112 mm Hg was considered as an hypotensive episode in the same patient.

During liver resection many events may result in arterial hypotension : bleeding, mobilisation of the liver, compression of the inferior vena cava and also the Pringle Manoeuvre itself or complete vascular exclusion of the liver. It would have been interesting to know if such procedures were used. In Table 3 the number of hypotensive episodes appears to be related to the time of operation, to the blood loss and to the duration of hypotensive episodes. Clearly all these factors are encountered in a difficult surgical procedure; more details concerning the surgical technique used would have been useful to interprete these data. Moreover concerning transfusion, whole blood transfusion was the single factor that significantly affected recurrence in univariate analysis. Usually such blood transfusions are administered in patients who actively bleed. This factor can also be considered as a reflection of the technical difficulty of liver resection.

Blood transfusion is known to affect post operative mortality in major liver surgery. It has also been suggested that it could affect the recurrence rate due to a potential immunosuppresive effect[4]. There is little information on the effect of intraoperative hemodynamic changes on the immune system.

The conclusion that perioperative bleeding and intraoperative hypotension should be avoided makes good sense. However the same criteria should be analysed in other large series with a sufficient follow-up before the prognostic value of these factors is taken for granted.

B. Nordlinger*, P. Balladur*, E. Delva**
Department of Surgery* and
Anesthesiology**
Hôpital Saint-Antoine
184 Fiubourg Saint-Antoine
75012 Paris, France

### REFERENCES

1. Doci, R., German, L., Bigmani, P., Montallo, F., Moranito, A. and Bozzetti, F. (1991) One hundred patients with hepatic metastases from colorectal cancer treated by resection : analysis of prognostic determinant *Br. J. Surg.*, **78**, 797–801
2. Hughes, K.S., Simon, R., Songhorabodi, S. *et al.* (1986) Resection of the liver for colorectal carcinoma metastase. A multi institutional study of patterns of recurrence. *Surgery*, **100**, 268–274

3. Scheele, J., Stangl, R., Altendorf-Hofman, A., Gall, F.P. *et al.* (1991) Indications of prognosis after hepatic resection for colorectal secondaries. *Surgery*, **110**, 13–23
4. Schriemer, P.A., Longnecker, D. and Mintz, P.D. (1989) The possible immunosuppression effects of perioperative blood transfusion in cancer patients. *Anesthesiology*, **68**, 422–428

### SHOULD NEITHER SCLEROTHERAPY NOR PROPRANOLOL BE USED PROPHYLACTICALLY FOR OESOPHAGEAL VARICES?

### ABSTRACT

*The PROVA Study Group. (1991) Prophylaxis of first hemorrhage from esophageal varices by sclerotherapy, propranolol or both in cirrhotic patients: A randomized multicenter trial. Hepatology;* **14**, *1016-1024*

**The objective of this randomized multicenter trial was to assess the prophylactic effect on the incidence and severity of the first variceal hemorrhage of endoscopic sclerotherapy, propranolol and the combination of the two compared with none of these treatments in patients with cirrhosis and esophageal varices. Among 819 cirrhotic patients who never had experienced variceal bleeding, esophagoscopy revealed varices in 379, of whom 286 were enrolled in the trial; 73 were allocated to sclerotherapy (paravenous polidocanol [10 mg/ml] every 1 to 2 wk until eradication), 68 to propranolol (slow-release preparation in one daily dose adjusted to provide about 25% heart rate reduction), 73 to both treatments and 72 to neither of the two treatments. The patients were observed for up to 42 months, with an average of 15 months. After variceal bleeding, patients in all groups received sclerotherapy only. The incidences of variceal bleeding ($n$ = 50) were almost identical in the four groups. The relative risk (with 95% confidence limits) with sclerotherapy was 1.06 (0.61 to 1.84), and the relative risk with propranolol was 0.92 (0.53 to 1.60). The mortality rate after variceal bleeding ($n$ = 29) did not differ significantly either. The mortality rate without variceal bleeding ($n$ = 46) was 2.75 (1.45 to 5.22) times higher in the sclerotherapy groups than in the nonsclerotherapy groups ($p$ = 0.002), whereas propranolol showed no effect, the relative risk being 1.17 (0.66 to 2.10). The total mortality rate showed no significant difference between the sclerotherapy, propranolol and control groups, but the combined therapy group had a significantly increased mortality rate.**

**This trial yielded evidence against prophylaxis of variceal hemorrhage in cirrhosis by endoscopic sclerosing injections, with or without propranolol and no support of propranolol used alone. (Hepatology 1991; 14:000-000.)**

